# Hypophosphatemia across the lifespan

**DOI:** 10.1093/jbmrpl/ziaf177

**Published:** 2025-12-06

**Authors:** Madhuni Herath, Richard Prince, Craig F Munns, Albert Kim

**Affiliations:** Departments of Endocrinology and Diabetes, Monash Health, Clayton, VIC 3168, Australia; Metabolic Bone Research Group, Centre for Endocrinology and Reproductive Health, Hudson Institute of Medical Research, Clayton, VIC 3168, Australia; Department of Medicine, School of Clinical Sciences, Monash University, Clayton, VIC 3800, Australia; Nutrition and Health Innovation Research Institute, School of Medical and Health Sciences, Edith Cowan University, Joondalup, WA 6027, Australia; Medical School, The University of Western Australia, Perth, WA 6009, Australia; Child Health Research Centre, Faculty of Health, Medicine and Behavioural Sciences, The University of Queensland, Brisbane, QLD 4072, Australia; Department of Endocrinology and Diabetes, Queensland Children’s Hospital, Brisbane, QLD 4101, Australia; Department of Diabetes and Endocrinology, Westmead Hospital, Sydney, NSW 2145, Australia; Faculty of Medicine and Health, University of Sydney, Sydney, NSW 2050, Australia

“Hypophosphatemia across the lifespan” is a concerted effort to highlight complex diagnosis and the systemic and bone-specific consequences of phosphate deficiency disease, as opposed to X-linked hypophosphatemia alone, to provide updated evidence for the clinical management of these patients while highlighting directions for future research. In 2020, the Therapeutics Committee of the Australian and New Zealand Bone Mineral Society (ANZBMS) established the Hypophosphatemia Interest Group under the leadership of Professor R.P., as an international and cross-discipline working party to improve care for affected patients. Professor C.F.M. led this group in publishing the Asia Pacific Consensus Guidelines in the management of X-linked hypophosphatemia (XLH), with a particular focus on a multidisciplinary approach and transitional care in supporting the affected individual from childhood through to adult life.^1^ This supplement is a celebration of this initiative and the impact of collaborative research in improving the lives of pediatric and adult patients affected by such underserved areas of medicine in Australia. Given the aim of considering hypophosphatemia in general as opposed to XLH alone several bone diseases, where hypophosphatemia is a critical pathophysiological feature are also discussed in this issue.

X-linked hypophosphatemia occurs secondary to an inactivating mutation of the phosphate regulating endopeptidase X-linked (PHEX) gene. This mutation subsequently leads to fibroblast growth factor 23 (FGF23) mediated abnormal renal handling of phosphate, clinically characterized by abnormal bone development, skeletal gait abnormalities, and importantly musculoskeletal symptomatology and other multi-system effects. Four papers consider specific aspects of XLH. Sandy et al. provide important insights into the impact of XLH from the perspective of individuals and caregiver.^2^ Hearing impairment that more commonly affects adults with XLH is less well appreciated; Oh et al. provide a comprehensive review of XLH-related auditory pathology.^3^

Fibroblast growth factor 23 is a key regulatory factor for phosphate homeostasis and is implicated in a range of hypophosphatemic bone disorders. Its discovery in 2000 was an important step toward changing the therapeutic landscape for individuals with FGF23-related bone disease. Building on this initial research, the advent of burosumab—a monoclonal antibody targeting FGF23—has changed the landscape of FGF23 mediated bone disorders, particularly XLH. Burosumab has made a profound impact on pediatric management of XLH. Sandy et al. provide complementary data and discussion on the acceptability of burosumab to pediatric patients and caregivers, important considerations for health care planning and delivery.^4^

In adults with established skeletal complications, the efficacy and benefits of burosumab are not as well established. Kumar et al. contribute the first Australian dataset to the handful of existing real-world studies on the impact of burosumab in adults, showing improved function and symptoms.^5^

While XLH secondary to a *PHEX* mutation is the most common genetic form of FGF23 mediated hypophosphatemic bone disease, other genetic forms have also been described and include autosomal dominant and autosomal recessive hypophosphatemic rickets. Collins et al. describe the clinical presentation and consequences of autosomal recessive hypophosphatemic rickets in neonates, caused by ENPP1 gene mutations, highlighting the importance of genetic testing in neonatal rickets.^6^

Given that there are several mechanisms through which hypophosphatemic bone disorders occur, both dependent and independent of FGF23, Park et al. outline a systematic approach and propose an algorithm for the evaluation of hypophosphatemia.^7^ This important paper should assist the clinician to navigate the complex diagnostic pathway. In addition, the case discussion provides evidence in the support of the benefits of burosumab in FGF23 dependent, non-PHEX mediated, likely genetic cause of hypophosphatemia and detail real-world experience of variability in patient response to therapy. This adds to the growing body of evidence supporting the use of FGF23 inhibition beyond XLH and emphasizes the importance of assessing FGF23 levels in relation to phosphate levels.

The impact of iatrogenic hypophosphatemia has been under-recognized. Dawson et al. characterize the scope of the issue through a retrospective review of patients treated with iron infusions, providing an analysis of severity and duration of hypophosphatemia and risk factors for developing the electrolyte abnormality.^8^ Iron polymaltose is commonly used in Australian hospitals due to its affordability and short infusion duration; however, the high incidence of hypophosphatemia with this particular formulation requires due consideration. Burrage et al. reported the incidence of hypophosphatemia and its timing in adult inpatients following iron carboxymaltose and polymaltose infusions.^9^

Particularly relevant to the Australian pharmacotherapeutic landscape is the impact of co-prescription of intravenous iron and subcutaneous denosumab. In Australia, prescription of denosumab increased to account for 76.1% of all prescriptions for bone antiresorptive therapy in 2018.^10^ Along with the frequent prescription of intravenous iron, particularly carboxymaltose formulation for the management of postoperative blood loss and iron deficiency, the population with fracture are at particularly increased risk of severe hypophosphatemia. Stokes et al. provide an in-depth discussion of the pathophysiology of this “double-trouble” and available evidence through review of a case series of affected patients in Sydney hospitals.^11^

With increased recognition and improved therapeutic options, the accurate diagnosis of hypophosphatemia is critical. The calculation of maximal tubular reabsorption of phosphate relative to glomerular filtration (TmP/GFR) is the initial diagnostic test to investigate hypophosphatemia, providing key information on renal handling of phosphate. Chiang et al. present an easy to use, online calculator culminating from collaboration between the AACB and ANZBMS to provide a validated tool for clinicians to utilize in diagnostic decision making.^12^

Taken together, this Special Issue of *JBMR Plus* highlights the diverse pathophysiological mechanisms and clinical manifestations of hypophosphatemia across the lifespan. The studies in this Issue reflect evolving insights into mechanisms, consequences and therapeutic advances of hypophosphatemia.
